# Response to anti-PD1 therapy with nivolumab in metastatic sarcomas

**DOI:** 10.1186/s13569-016-0064-0

**Published:** 2016-12-30

**Authors:** L. Paoluzzi, A. Cacavio, M. Ghesani, A. Karambelkar, A. Rapkiewicz, J. Weber, G. Rosen

**Affiliations:** 1 Department of Medicine, NYU Langone Medical Center, New York, NY USA; 2 Department of Radiology, NYU Langone Medical Center, New York, NY USA; 3 Department of Pathology, New York University School of Medicine, Laura and Isaac Perlmutter Cancer Center, 10th floor, Room 1041, 160 East 34th street, New York, NY USA

**Keywords:** Sarcoma, Immunotherapy, Nivolumab, PD-1, Check point inhibitors

## Abstract

**Background:**

Manipulation of immune checkpoints such as CTLA4 or PD-1 with targeted antibodies has recently emerged as an effective anticancer strategy in multiple malignancies. Sarcomas are a heterogeneous group of diseases in need of more effective treatments. Different subtypes of soft tissue and bone sarcomas have been shown to express PD-1 ligand.

**Methods:**

We retrospectively analyzed a cohort of patients (pts) with relapsed metastatic/unresectable sarcomas, who were treated with nivolumab provided under a patient assistance program from the manufacturer. Pts underwent CT or PET/CT imaging at baseline and after at least four doses of nivolumab; RECIST 1.1 criteria were used for response assessment.

**Results:**

Twenty-eight pts with soft tissue (STS, N = 24) or bone sarcoma (N = 4), received IV nivolumab 3 mg/kg every 2 weeks from July 2015. Median age was 57 (24–78), male:female ratio was 14:14; the median number of nivolumab cycles was eight. Eighteen pts concomitantly received pazopanib at 400–800 mg daily. The most common side effect was grade 1–2 LFT elevations; grade 3–4 toxicity occurred in five patients (colitis, LFT elevations, pneumonitis). Twenty-four pts received at least four cycles. We observed three partial responses: one dedifferentiated chondrosarcoma, one epithelioid sarcoma and one maxillary osteosarcoma (last two patients on pazopanib); nine patients had stable disease including three leiomyosarcomas; 12 patients had progression of disease including 4 leiomyosarcoma. Clinical benefit (response + stability) was observed in 50% of the evaluable patients.

**Conclusions:**

These data provide a rationale for further exploring the efficacy of nivolumab and other checkpoint inhibitors in soft tissue and bone sarcoma.

**Electronic supplementary material:**

The online version of this article (doi:10.1186/s13569-016-0064-0) contains supplementary material, which is available to authorized users.

## Background

Soft tissue (STS) and bone sarcomas are a heterogeneous group of diseases with an estimated 15,000 new cases in the US in 2016, and more than 50 different subtypes [1, 2]. Given their rarity and diversity, enrollment into prospective clinical trials has been very challenging, even in the context of cooperative groups. Despite new studies elucidating the genomic basis and the sensitivity to chemotherapy of specific subtypes, the overall prognosis of patients with metastatic sarcoma remains poor in most cases. Since 2012, new drugs such as pazopanib, and for more specific subtypes, trabectedin and eribulin, have been approved for patients who have relapsed after front line chemotherapy, but the response rates for these agents remains suboptimal [3]. Immunotherapy has recently provoked great interest in oncology after phase III clinical trials have shown significant efficacy in chemotherapy-resistant malignancies such as metastatic melanoma or renal cell carcinoma, and activity in chemotherapy-sensitive histologies including non-small cell lung cancer, head and neck cancer, MSI-high colon cancer and Hodgkin’s lymphoma.

Programmed death 1 (PD-1) is a surface receptor expressed on activated and exhausted T cells, which mediates inhibition of activation, cytokine secretion and lytic activity upon binding with its ligands (PD-L1 and PD-L2). The role of the PD-1/PDL-1 axis in suppression of T cell activation and its targeting through specific monoclonal antibodies, has been the basis for the success achieved in a number of clinical trials [4]. Tumor PD-L1 expression has been reported in up to 65% of different subtypes of sarcomas [5]. The degree of PD-1 positivity in tumor-infiltrating lymphocytes (TILs) and PD-L1 expression in tumor specimens from 105 cases of soft tissue sarcomas, has been correlated with a poorer prognosis and more aggressive disease [6]. While preclinical studies and retrospective analyses of clinical data may provide a rationale for immune-mediated strategies against sarcoma, there are currently very limited clinical data to support the use of anti-PD-1 antibodies in this setting. We report herein a retrospective series of twenty-eight patients with metastatic or locally advanced soft tissue or bone sarcoma who received the PD-1 antibody nivolumab under a patient assistant program off protocol, with or without pazopanib. We describe for the first time clinical benefit in different subtypes, as shown by disease regression or stabilization.

### Patients and methods

Between July 2015 and August 2016, twenty-eight patients with a diagnosis of soft tissue or bone sarcoma were treated with nivolumab. All patients but two, previously received one line of systemic treatment; patients receiving pazopanib before starting nivolumab were continued on this treatment given concern for disease flare after discontinuation, as described for other tyrosine kinase inhibitors [7]. The following data were recorded for all patients: gender, age, location of the primary sarcoma, stage, median number of prior therapies, ECOG performance status, number of cycles of nivolumab administered. Nivolumab was given at the standard dose of 3 mg/kg IV every 2 weeks; the drug was provided by the manufacturer under a patient assistant program. Complete blood count, electrolytes, liver and kidney function tests were performed before each cycle of treatment, more often if clinically indicated; all toxicities were recorded at each visit (at least every 2 weeks), as per NCI CTCAE v.4.0. Baseline scans consisted of PET/CT or CT scans with IVC, imaging was repeated every two or three months. Next generation sequencing to determine the presence of specific mutations in a panel of 50 genes was performed in one patient with a dedifferentiated chondrosarcoma responding to nivolumab alone; the polymerase chain reaction (PCR) product for specific mutations was sequenced on an Ion Torrent PGM instrument (Thermo Fisher Scientific, Waltham, MA). PD-L1 expression was assessed in selected patients who had evaluable tissue for testing (N = 10) and performed at Esoterix Genetic Laboratories (Integrated Oncology, New York, NY). PD-L1 positive was defined as a tumor proportion score (% of at least 100 viable tumor cells with complete or partial 1 + membrane staining) of 50% for at least 100 viable tumor cells exhibiting membrane staining. For PD-L1 detection, we used the PD-L1 IHC 28-8 pharmDx assay (Dako North America Inc, CA, USA).

## Results

### Patients and treatment

The clinical characteristics of the patients are shown in Table 1. A total of 28 patients with a diagnosis of metastatic (n = 26) or unresectable (n = 2) soft tissue or bone sarcoma received IV nivolumab every 2 weeks; median age was 57 years, female to male ratio was 14:14, ECOG performance status was 0–1 for 24 patients, and 2 for the remaining 4 patients. Eighteen patients received concomitant pazopanib. The median number of prior systemic treatments, including neoadjuvant and adjuvant chemotherapy was 2 (range 0–6). Sarcoma subtypes are listed in Additional file 1: Table S1; twenty-four patients had a diagnosis of a soft tissue sarcoma with leiomyosarcoma (LMS) being the most common subtype (n = 7); four patients had conventional osteosarcoma (OS).

**Table 1:** Patient baseline characteristics

Factor	No
Age	
Median	57
Range	24–78
Sex	
Female	14
Male	14
Soft tissue sarcoma	24
Bone sarcoma	4
Location	
Extremity	6
Abdomen/pelvis	12
Axial	2
Head/neck	5
Chest	3
Stage	
IV	26
Unresectable	2
ECOG PS	
0–1	24
2	4
Nivolumab	
Cycles (median)	8
Range	1–26
On Pazopanib	18
Prior treatments (including neoadjuvant/adjuvant)	
Median	2
Range	0–6
Anthracycline	15
Ifosfamide	10
Gemcitabine	9
Docetaxel	7

### Safety

Table 2 shows all the adverse events (AE); most side effects were grade 1–2 with a predominance of LFT abnormalities (8 out of 10 patients on pazopanib). Grade 3–4 AE were experienced by four patients, all on pazopanib. One patient experienced grade 3 elevation of AST/ALT/alkaline phosphatase and grade 4 bilirubin elevation after two cycles of nivolumab; liver biopsy was consistent with drug related hepatitis; she discontinued treatment with normalization of bilirubin, and improvement of ALT/AST to grade 1. A second patient experienced grade 3 ALT elevation that improved to grade 1 once both nivolumab and pazopanib were discontinued and high dose steroids (prednisone 1 mg/kg/daily) were administered with a slow taper over about 2 months; she subsequently restarted treatment with both drugs. A third patient had grade 4 AST/grade 3 ALT/alkaline phosphatase elevations with grade 3 pneumonitis that required intubation; he recovered after high dose steroids and he was able to restart pazopanib only, after LFTs normalized. A fourth patient experienced grade 3 colitis that significantly improved with high dose steroids; she was restarted on treatment with both pazopanib and nivolumab until she progressed.

**Table 2:** Safety

	Grade 1–2			Grade 3–4		
	On Pz (No.)	NO Pz (No.)	Total (No.)	On Pz (No.)	NO Pz (No.)	Total (No.)
Hematologic						
Anemia	3	1	4	–	–	–
Neutropenia	1	–	1	–	–	–
Thrombocytopenia	1	1	2	–	–	–
Non-hematologic						
Diarrhea	3		3	1		1
Pneumonitis	1		1	1		1
Rash	3	1	4	–	–	–
Hypothyroidism	6	2	8	–	–	–
LFTs	8	2	10	3		3

Toxicity was graded as per NCI CTCAE v4.0

*Pz* pazopanib, 400–800 mg po daily; *LFTs* liver function tests abnormalities

### Efficacy outcomes

**Fig. 1:**
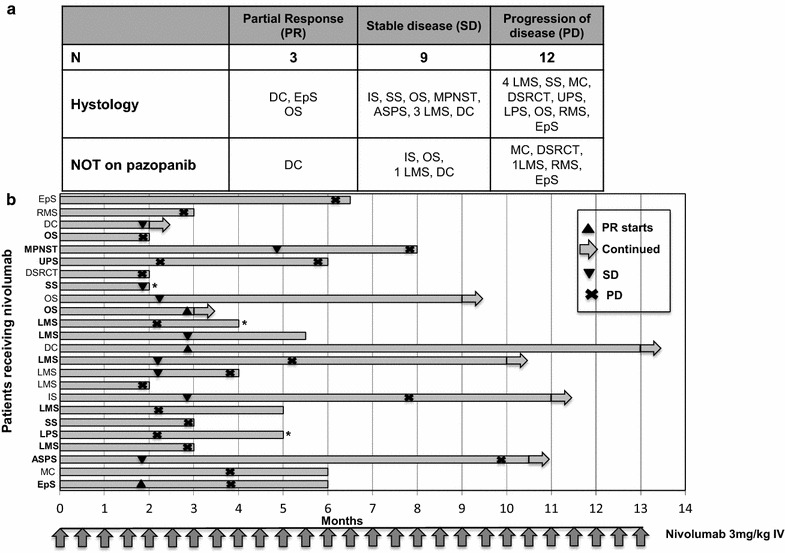
Response assessment after nivolumab. **a** Best responses; sarcoma subtypes and concomitant use of pazopanib are shown. **b** Swimmer plot in 24 patients who received at least four doses of nivolumab. Patients on pazopanib are indicated in bold on the *Y axis* with the correspondent histology. *DC* dedifferentiate chondrosarcoma; *EpS* epithelioid sarcoma; *MC* mesencymal chondrosarcoma; *LPS* liposarcoma; *LMS* leiomyosarcoma; *ASPS* alveolar soft part sarcoma; *SS* synovial sarcoma; *IS* intimal sarcoma; *OS* osteosarcoma; *DSRCT* desmoplastic small round cell tumor; *MPNST* malignant peripheral nerve sheet tumor; *UPS* undifferentiated pleomorphic sarcoma; *RMS* rhabdomyosarcoma. *Patient died

**Fig. 2:**
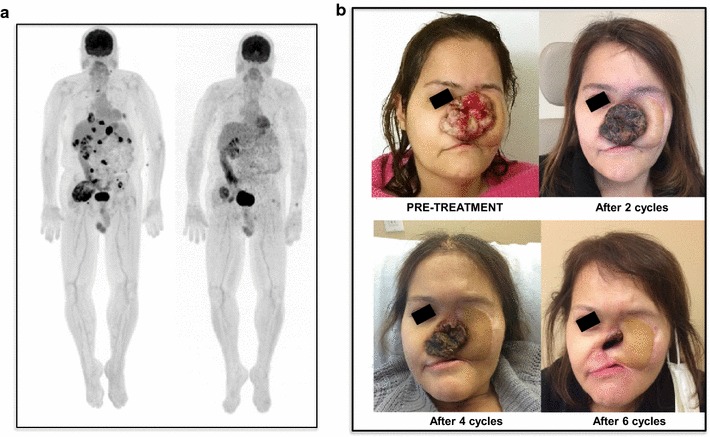
Partial response (PR) to nivolumab in 2 patients. **a** PET/CT of a 74 year-old male with metastatic dedifferentiated chondrosarcoma after six cycles of nivolumab alone; he is maintaining a PR after 26 cycles. **b** 46 years-old woman with osteosarcoma, treated with nivolumab for six cycles; pazopanib 800 mg p.o. daily was started after 4 cycles of nivolumab. She underwent resection with negative margins

Twenty-four patients were evaluable for response (Fig. 1). Four patients were not evaluable for the following reasons: liver toxicity after 2 cycles (n = 1); patients lost at follow up (n = 2), concomitant radiation therapy (n = 1). We observed three partial responses (PR) and they included: a 74 year-old patient with a dedifferentiated chondrosarcoma (DC), after six cycles of nivolumab alone with PR maintained after 26 cycles (Fig. 2a; Additional file 1: Figure S1). The NGS-tumor 50 panel only showed non-synonymous variants of unknown significance for the PIK3CA and TP53 genes; PD-L1 staining was 20%. A second PR was observed in a 46 year-old female with a relapsed, OS of the left maxilla. She had a minimal clinical response to nivolumab given for four cycles; pazopanib was then added and it was given for only one month (Fig. 2b). The rationale to add pazopanib after 4 cycles of nivolumab alone, relied on the following considerations: (1) the original lesion showed abundant vascularization; (2) pazopanib targets the vascular endothelial growth receptors VEGF-1, VEGFR-2, VEGF-3; (3) nivolumab alone was tolerated very well; (4) a resection of this challenging lesion could potentially give the best chance for a long progression free survival in this young patient. After one month of pazopanib, her facial lesion significantly regressed and the patient had major clinical benefit in terms of improved eating habits and pain control. At that point we thought it was in the patient’s best interest to undergo surgery. At the time of resection, the tumor showed extensive necrosis and margins were negative. PD-L1 in this patient was <5%. A third PR was observed in a 24 year-old man with a proximal type epithelioid sarcoma (EpS) metastatic to the lung progressing on pazopanib. We decided to continue pazopanib given the concern for disease flare after discontinuation as described for other tyrosine kinase inhibitors [7]. This patient had a PR after four cycles of nivolumab, progression (PD) due to a new lesion in the left lung after four additional cycles; he had further PD in the lung after four more cycles and nivolumab was stopped.

Nine patients had stable disease (SD): five patients received pazopanib plus nivolumab while four patients received nivolumab alone (one dedifferentiated chondrosarcoma, one leiomyosarcoma, one intimal sarcoma and one osteosarcoma).

A patient with an alveolar soft part sarcoma (ASPS) progressing on pazopanib, with a slight PD (~25% increase per RECIST criteria) after nine cycles of nivolumab, he is overall asymptomatic and continues treatment with both drugs; two patients with uterine LMS, both progressing on pazopanib alone, one had SD after five cycles (PD after 6 more) and the other SD after six cycles (nivolumab stopped after five more cycles because of pneumonitis); a patient with a LMS of the vulva had SD after five cycles of nivolumab alone but a PD after three additional cycles; a patient with an intimal sarcoma (IS) of the right pulmonary artery metastatic to both lungs, experienced a SD after six cycles of nivolumab alone (the primary site was not evaluable because previously irradiated); he had PD after six additional cycles but continues nivolumab because he is asymptomatic; a patient with a synovial sarcoma (SS) had a SD after four cycles, but died from complications during surgery for repair of a pulmonary artery pseudo-aneurysm; a patient with a maxillary OS had SD after five cycles of nivolumab alone (confirmed after a total of 16 cycles); a patient with a malignant peripheral sheet tumor (MPNST), on pazopanib, had stability after four cycles but progressed after six additional cycles of nivolumab; a patient with a dedifferentiated myxoid chondrosarcoma (MC) had SD after four cycles of nivolumab.

Overall, 12 out of 24 evaluable patients had clinical benefit (PR + SD). Twelve patients had PD: four patients with LMS (three on pazopanib): one transferred care to another hospital after six cycles, one had PD after five cycles, confirmed after five more, and switched to another treatment, one received five cycles and died after an accidental fall, a last one had a PD after four cycles on nivolumab alone. Additional PD in patients on pazopanib included: one with a MC after four cycles (confirmed after 4 more), one with a liposarcoma (LPS) after five cycles (confirmed after 6 more), one with an OS after four cycles; a patient with a SS after six cycles complicated by severe pneumonitis; one patient with an undifferentiated pleomorphic sarcoma (UPS) of the right upper extremity after five cycles (confirmed after 7 more). Patient with PD on nivolumab alone included: one with a desmoplastic small round cell tumor (DSRCT) after four cycles; one patient with a rhabdomyosarcoma (RMS) after six cycles; one with an EpS after 13 cycles.

## Discussion

Patients with metastatic soft tissue sarcomas generally have a poor prognosis, with low response rates after first line chemotherapy [3]. Of note, the tyrosine kinase inhibitor pazopanib was approved by the FDA in 2012 on the basis of a phase III randomized trial showing improved PFS in the second line setting; the overall response rate was only 6% [8].

Multiple recent genomic studies have provided better insight into sarcoma biology through a more accurate classification by molecular subtype, identification of recurrent mutations in oncogenic pathways and evidence of epigenetic dysregulation [9]. Barretina et al. [10] for example, recently provided a comprehensive database of sarcoma genome alterations in 207 samples of STS; despite their elucidation of genes and signaling pathways not previously associated with STS, we still lack appropriate pharmacologic tools for targeting specific genomic alterations.

Several subtypes of STS are characterized by specific chromosomal translocations which result in unique fusion proteins; while many of them function as transcription factors, making their therapeutic targeting quite challenging, these proteins may represent attractive targets from an immunotherapy standpoint [11]. Immunogenicity of sarcoma is supported by several preclinical studies and some clinical data with human sarcoma specimens.

Immunotherapeutic strategies in sarcoma have included cytokine-based immunotherapies, treatment with muramyl tripeptide phosphatidyl ethanolamine in osteosarcoma, vaccines and adoptive immunotherapy to cite a few examples, although none have appeared promising to date [12]. Our retrospective analysis shows the potential clinical benefit from treatment of soft tissue and bone sarcomas with the anti-PD1 antibody nivolumab.

This is not a prospective study, and given the retrospective nature of this series, it has several limitations; data on patients who received either nivolumab alone (N = 10) or nivolumab + pazopanib (N = 18), were pulled together in order to capture a possible signal of activity from immunotherapy (alone or in combination) that may be helpful for a following prospective study. Additionally, this is small study with multiple hystologies included: the largest group of patient had a diagnosis of leiomyosarcoma (N = 7), but most subtypes are represented by only 1 or 2 patients.

In our series we showed disease improvement or stabilization in 12/24 patients evaluable for response. Eighteen out of twenty-eight patients concomitantly received pazopanib, however 1 partial response was observed in a dedifferentiated chondrosarcoma on nivolumab alone. Another response was seen in a patient with an unresectable maxillary OS who received four cycles of nivolumab and only one month of pazopanib that was started after the 4 cycles of nivolumab. A third patient with an epithelioid sarcoma, progressing on pazopanib, had a partial response after only four cycles of nivolumab; unfortunately he progressed after four additional cycles. Of note, overall responses were observed in some subtypes that are generally resistant to traditional chemotherapy such as dedifferentiated chondrosarcoma and epithelioid sarcoma. Interestingly, all the three aforementioned patients received adjuvant radiation therapy up to 20 years before, bringing up the possibility of a distant abscopal effect as hypothesized for other diseases such as melanoma [13].

At least three prospective phase II studies are exploring the role of the checkpoint inhibitors pembrolizumab and nivolumab in metastatic STS/bone sarcomas and/or uterine LMS; preliminary data were recently presented at the ASCO 2016 conference for two studies. Pembrolizumab showed some interesting responses in undifferentiated pleomorphic sarcoma (4/9), liposarcoma (2/9), synovial (1/9), chondrosarcoma (1/6) and osteosarcoma (1/19); no responses were seen in LMS (0/10) and Ewing sarcoma (1/13) [14]. Interestingly in our series, we also observed a partial response in one patient with a dedifferentiated chondrosarcoma and a PD-L1 expression that was higher compared to all other tested patients (20% versus less than 5%); additionally, Kostine et al. [15] recently showed that this specific subtype of bone sarcoma expresses PD-L1 in association with immune-infiltrating cells and HLA class I in nearly 50% of cells. Immunotherapy with check-point inhibitors seems a particularly promising approach for the treatment of this rare and challenging histology but more data is needed.

A second prospective study is exploring nivolumab in 12 patients with uterine LMS and showed no responses [16]. In our series, among seven patients with LMS we observed 4 PD and 3 SD. LMS is characterized by a significant degree of morphologic and molecular heterogeneity and different molecular subtypes may respond differently to immunotherapy [17].

The combination of nivolumab and pazopanib is interesting but needs dose optimization to prevent, in particular, excessive liver toxicity. Nivolumab at 2 mg/kg every 3 weeks has been combined with pazopanib at 800 mg po daily in patients with renal cell carcinoma; about 70% of patients experienced grade 3–4 side effects, mainly LFT abnormalities, fatigue and diarrhea [18].

## Conclusion

We describe a cohort of 28 sarcoma patients with metastatic or unresectable soft tissue or bone sarcomas who were treated with the anti-PD-1 antibody nivolumab with or without the tyrosine kinase inhibitor pazopanib; we found evidence of clinical benefit with a half of the evaluable patients experiencing a partial response or a stabilization of disease after at least 4 cycles of nivolumab. Given the potential activity of nivolumab alone and promising data when combined with pazopanib, we are planning a prospective, phase II randomized study of nivolumab alone versus nivolumab with pazopanib, in metastatic soft tissue and bone sarcomas; correlative studies will include tumor and serum sampling for correlation with the clinical endpoints of response and progression-free survival.

## Additional file

**Additional file 1.** **Table S1.** Sarcoma subtypes. Primary site and concomitant treatment with pazopanib given at 400-800 mg po daily, are indicated. DC = dedifferentiate chondrosarcoma; MC = mesenchymal chondrosarcoma; LPS-DD = dedifferentiated liposarcoma; LMS = leiomyosarcoma; ASPS = alveolar soft part sarcoma; SS = synovial sarcoma; EpS = epithelioid sarcoma; IS = intimal sarcoma; UPS = undifferentiated pleomorphic sarcoma; DFSP = dermatofibrosarcoma protuberans; OS = osteosarcoma: MPNST = malignant peripheral nerve sheet tumor; DSRCT = desmoplastic small round cell tumor; RP = retroperitoneum; UE = upper extremity; LE = lower extremity. **Figure S1.** Partial response (PR) to nivolumab in a 74 year-old male with metastatic dedifferentiated chondrosarcoma. This patient received 6 cycles of IV nivolumab 3 mg/kg every 2 weeks; he is maintaining a PR after 26 cycles.

## Abbreviations

pts: patients
STS: soft tissue sarcoma
PD-1: programmed death 1
PDL-1: programmed death ligand 1
PCR: polymerase chain reaction
TILs: tumor infiltrating lymphocytes
LFT: liver function tests
AST: aspartate transaminase
ALT: alanine transaminase
LMS: leiomyosarcoma
OS: osteosarcoma
DC: dedifferentiated chondrosarcoma
EpS: epithelioid sarcoma
IS: intimal sarcoma
SS: synovial sarcoma
MPNST: malignant peripheral nerve sheet tumor
MC: mesenchymal chondrosarcoma
LPS: liposarcoma
UPS: undifferentiated pleomorphic sarcoma
DSRCT: desmoplastic small round cell tumor
RMS: rhabdomyosarcoma
ASPS: alveolar soft part sarcoma
AE: adverse event
PD: progression of disease
SD: stable disease
PR: partial response
PFS: progression free survival
CTLs: cytotoxic T-lymphocytes
