# Supplementary material for: Response to anti-PD1 therapy with nivolumab in metastatic sarcomas
Source: Clin Sarcoma Res. 2016 Dec 30;6:24. doi: 10.1186/s13569-016-0064-0 (PMC5200964; doi:10.1186/s13569-016-0064-0)
Supplement: Supplementary file 1 — Additional file 1. Table S1. Sarcoma subtypes. Primary site and concomitant treatment with pazopanib given at 400-800 mg po daily, are indicated. DC = dedifferentiate chondrosarcoma; MC = mesenchymal chondrosarcoma; LPS-DD = dedifferentiated liposarcoma; LMS = leiomyosarcoma; ASPS = alveolar soft part sarcoma; SS = synovial sarcoma; EpS = epithelioid sarcoma; IS = intimal sarcoma; UPS = undifferentiated pleomorphic sarcoma; DFSP = dermatofibrosarcoma protuberans; OS = osteosarcoma: MPNST = malignant peripheral nerve sheet tumor; DSRCT = desmoplastic small round cell tumor; RP = retroperitoneum; UE = upper extremity; LE = lower extremity. Figure S1. Partial response (PR) to nivolumab in a 74 year-old male with metastatic dedifferentiated chondrosarcoma. This patient received 6 cycles of IV nivolumab 3 mg/kg every 2 weeks; he is maintaining a PR after 26 cycles. [file 13569_2016_64_MOESM1_ESM.pdf]

Subtype	No.	Primary site (No.)	Concomitant Pazopanib
LMS	7	uterus (4), vulva (2), RP (1)	yes (4), no (3)
OS	4	pelvis (1), maxilla (2), humerus (1)	yes (3), no (1)
UPS	3	quadratus lomborum, UE (2)	yes
SS	2	UE, LE	yes
EpS	2	neck, chest wall	yes (1), no (1)
MC	1	skull	no
DC	2	buttock, LE	no
IS	1	pulmonary artery	no
DFSP	1	scalp	yes
MPNST	1	lumbar spine	yes
LPS-DD	1	RP	yes
ASPS	1	chest wall	yes
DSRCT	1	adrenal	no
RMS	1	diaphragm	no

Supplementary Table 1. Sarcoma subtypes. Primary site and concomitant treatment with pazopanib given at 400-800mg po daily, are indicated.
DC= dedifferentiate chondrosarcoma; MC= mesenchymal chondrosarcoma; LPS-DD=dedifferentiated liposarcoma; LMS=leiomyosarcoma; ASPS=alveolar soft part sarcoma; SS=synovial sarcoma; EpS=epithelioid sarcoma; IS=intimal sarcoma; UPS=undifferentiated pleomorphic sarcoma; DFSP=dermatofibrosarcoma protuberans; OS=osteosarcoma: MPNST=malignant peripheral nerve sheet tumor; DSRCT=desmoplastic small round cell tumor; RP=retroperitoneum; UE=upper extremity; LE=lower extremity.

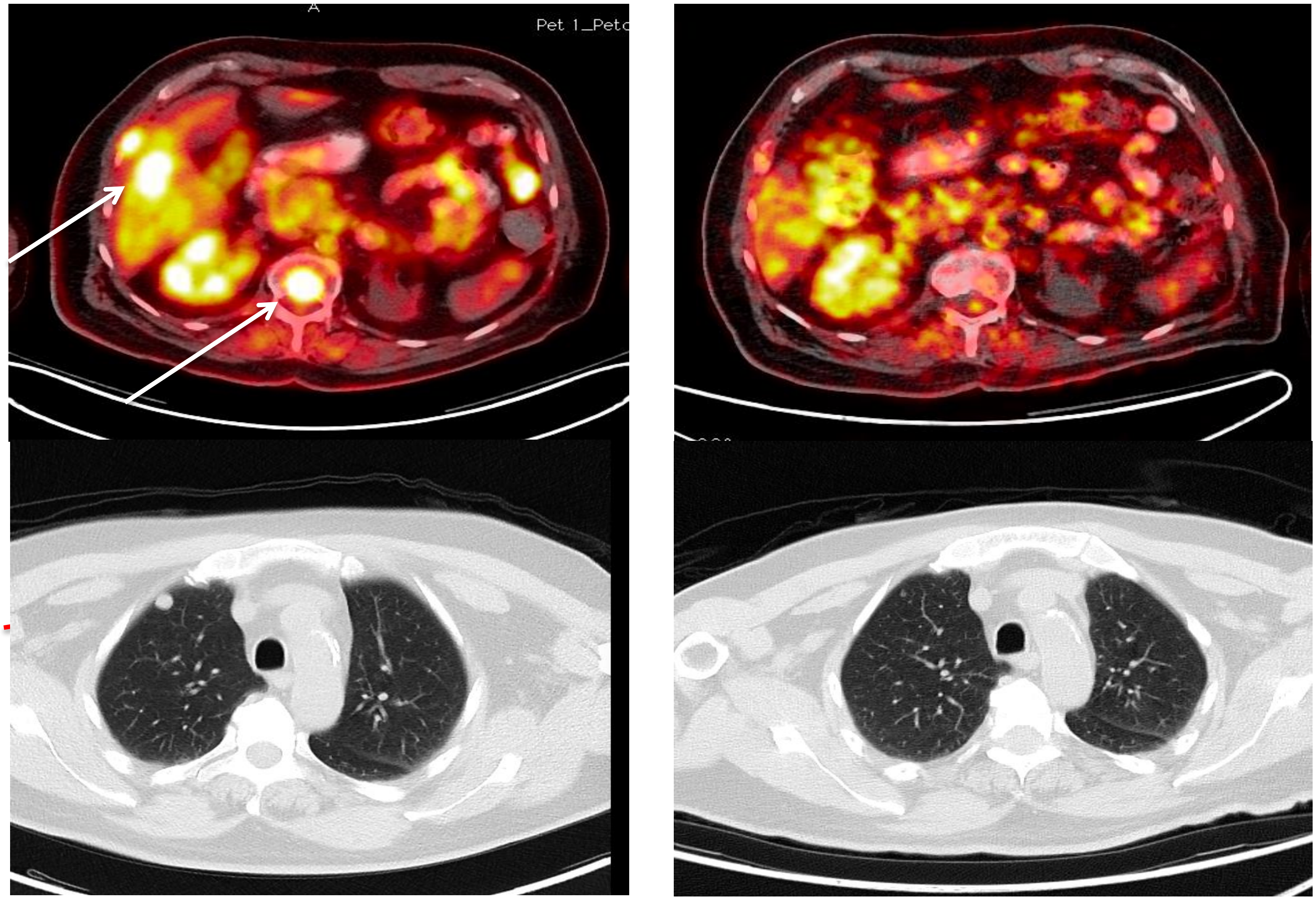

PRE-TREATMENT After 6 cycles

Supplementary Figure 1. Partial response (PR) to nivolumab in a 74 year-old male with metastatic dedifferentiated chondrosarcoma. This patient received 6 cycles of IV nivolumab 3mg/kg every 2 weeks; he is maintaining a PR after 26 cycles.
